# Letter To The Editor: The challenges faced by providers of CME in Europe

**DOI:** 10.1080/21614083.2017.1350929

**Published:** 2017-07-18

**Authors:** Sophie Wilson

**Affiliations:** ^a^ gCMEp group Chair and International Medical Press, London, UK

Dear Editor

It is widely acknowledged that there is an increasing need to uphold high standards of medical education in Europe. I write on behalf of the Good CME Practice group (gCMEp), to share our concerns around the definition of Continuing Medical Education (CME) and its practice in Europe. We believe there is a need to clarify the differences between the various types of continuing education in the health professions.

In the absence of clear and consistent guidance from accreditation agencies, and the emerging guidance from industry regulatory bodies in Europe, providers are faced with conflicting requirements for independence, accreditation and use of funding. Frequently, definitions and guidelines from the accreditation and industry bodies are in direct opposition to each another.

At the recent 9th Annual European CME Forum [1] there were presentations from various relevant parties including industry. In one such workshop, MedTech Europe, representing the medical devices and diagnostics industry, introduced their new standards in which their member organisations are no longer permitted to develop and present therapy area or “non-promotional”, education beginning in 2017. This type of education has become popular in recent years among pharmaceutical and medtech companies, where, rather than product-specific promotion, the company initiates and controls, and even develops and presents balanced education in the therapy area in general. While there are attractions to both the company and the learners, the lack of independence leads to an unresolvable problem of bias.

At the workshop, a consensus view was advanced with the approval of both the International Pharmaceutical Alliance for CME (iPACME) and the European Federation of Pharmaceutical Industries and Associations (EFPIA). Industry-supported education by means of independent grants and which is CME-accreditable should be described as independent medical education (IME). IME activities must be independent, fair and balanced without influence or input from the supporter, as such if the provider submits and succeeds in getting the activity accredited, then the IME would be considered as CME. The other three types of education supported by industry are (a) industry-controlled product specific educational programmes, (b) industry-initiated professional development/medical disease programmes (of the type forbidden by their MedTech counterparts discussed above) and (c) collaborative partnerships. None of these varieties of education is accreditable for CME and they do not qualify for the description of IME, but are all permitted by the pharmaceutical industry.

These discussions are welcome steps in the right direction. However, it is important to consider the voice of the providers, as they are independent and experienced international educators who bring an additional perspective allowing for a complete picture of IME/CME.

The gCMEp group has identified a need to develop their definition of what makes “good” IME/CME in the European arena. The gCMEp definition is based on the four core principles published by the group [2], and which should form the cornerstone of all good IME/CME programmes. We believe these principles underpin good CME across all regulatory and cultural borders throughout Europe. Today, more than ever, it is important to adhere to these principles as challenges are faced by all parties including, but not only providers, funders, societies and accrediting bodies.

## The gCMEp group’s four core principles

### Appropriate education

To decide what type of medical education is most effective in each situation, not only depends upon the particular context or target audience, but also on judgments made logically and scientifically in accordance with theories of learning and instruction. This enables gCMEp group members to identify which educational initiative is most likely to produce the desired results with a given group of healthcare professionals (HCPs). When planning appropriate medical education, five elements are fundamental in the design process. They can be summarised by answers to these questions:

- Is there a gap in clinical knowledge, competence and performance, and need for instruction? (gap analysis, needs assessment)
- For whom should the programme be developed? (characteristics of learners, target audience)
- What should the learners/HCPs learn or demonstrate? (learning objectives)
- How is the medical subject content or skill best learned? (educational strategies)
- How is the extent to which learning is achieved determined? (outcomes measurement, evaluation procedures)

Appropriate education, as defined by the gCMEp group, is created using a clear set of measurable learning objectives that are derived from a needs assessment, which identifies gaps in the HCP’s knowledge, competence and/or performance. The education must address the identified gaps by aligning with the needs of the learners, the learning objectives, and be based on adult learning principles. The educational activity can be measured to determine its impact and modified when necessary. The goal of the education is to elicit and reinforce best clinical practice in the healthcare setting, and ultimately improve patient outcomes.

### Effective education

To demonstrate the effectiveness of education, the gCMEp group subscribes to the practice of outcomes measurement and utilises the outcomes framework for planning and assessing CME described by Moore et al. [3]. At a minimum, level 3 (knowledge) should be achieved and measured. Depending on the needs of the learners, where possible, levels 4 (competence) and above (level 5 – performance, level 6 – patient health, level 7 – community health) should be achieved and measured. Programmes that aim for levels 4 and higher require careful planning and collaboration with an extended planning committee. This includes, but is not limited to, physicians and other HCPs, healthcare systems, allied healthcare providers, patients and carers.

While traditional didactic lectures and hour-long programmes have their place, there are many additional types of education that are effective, measurable and have a positive impact on patient care. The educational needs of physicians are constantly changing due to innovation and rapid advances in technology. The gCMEp group calls upon the accreditation bodies in Europe to examine their practices of accreditation, and to consider accrediting programmes that are effective, measure higher level outcomes and meet the rapidly changing education and practice needs of today’s physician.

### Transparent education

Transparent education means not only doing the right thing, but showing that it is being done both ethically and honestly; it is a question of public trust and accountability. All relevant information should be disclosed to the learners prior to, and during, the educational activity so that they understand fully how the content has been developed and presented. Best practices for transparency include:

- Documentation of the independent planning process
- Disclosure in marketing, logistical, and educational content of all financial support received for the programme (supporter name and role, but no marketing logos)
- Disclosure in marketing, logistical, and educational content of all relevant relationships of those influencing content (planning committee, faculty, organisers, etc.)
- Reporting, as necessary, to CME/CPD accreditors, industry supporters, or other regulatory bodies

### Balance

To meet the requirements of good CME, all content must be balanced. In a similar way to the transparency principle, this allows learners to trust that the educational activity delivers the full picture of the educational objective and not a one-sided view. To ensure balance in the programme, the following criteria must be met:

- All content must be reviewed and controlled by the designated faculty
- Content must be evidence-based
- Content must not be influenced by industry supporters or any other organisation that may have a perceived agenda
- Potential conflicts of interest for all faculty and people involved in the scientific content of the educational activity (e.g. members of the planning committee) must be identified and resolved
  - Resolution of any perceived conflicts of interest may include a provision of peer review by the programme chair or other faculty members
- All disclosure information must be communicated to learners

In order to meet the above criteria for balance, the gCMEp group acknowledges that all funding of IME/CME programmes should come from independent or arms-length funding. This is encouraged by groups within the pharmaceutical industry, who are working to define and clarify the role of the funder in offering financial support. Traditionally, in the medical sector this has been referred to as an “unrestricted educational grant”, which is a term that is now anachronistic and unhelpful, as under current accountancy definitions and practices, all financial transactions must have a clearly defined legal purpose or pre-defined “restrictions” defining the true independence of the funding. The preferred term is therefore “independent grant” and accreditation bodies and industry must clarify the difference between such funding, where no benefits to the supporter are allowed, and “sponsorship”, which is unsuitable for direct funding of IME/CME programmes as the supporter as “sponsor” may receive tangible commercial benefit in return for the funding.

The gCMEp group advocates that a clear distinction be made by all parties between education providers who develop education by means of grant funding independently of industry control and Medical Communications (Med Comms), PR and advertising agencies, who work on behalf of industry, being governed by their regulatory and legal obligations which require review and control of all content by their industry “clients”.

## Conclusion

The role of the provider in the provision of quality IME/CME is becoming increasingly important and thus the provider’s role must continue to be better defined in Europe. While some other countries, most notably the USA and Canada, have clearly defined the provider’s role, it is still evolving in Europe. It is the opinion of the gCMEp group that now is the time to bring clarity and definition to the role of the European provider in IME/CME. It is important to have continuing dialogue with key stakeholders, including industry, accreditation bodies and scientific societies to ensure the adoption of a clear, pragmatic approach to IME/CME that consistently delivers effective high-quality programmes for European physicians and other healthcare professionals; programmes should be funded, developed and presented appropriately, with the goal of improving patient care and outcomes.

The gCMEp group is carrying out an internal review of the practice of its members; as well as a review of educational best practices, industry standards and accreditation body expectations. We began communicating these practices at the 9^th^ European CME Forum in November 2016 and are committed to refining and sharing them with the IME/CME community. The gCMEp group is currently developing its recommendations regarding further guidance on the role of the provider within the IME/CME community and practical methods of applying the Four Core Principles of Good CME. We will be publishing a paper addressing this in Autumn 2017.
